# NSAID-Associated Perforation of a Meckel’s Diverticulum: A Case Report

**DOI:** 10.4021/jocmr504w

**Published:** 2011-04-04

**Authors:** Avneet S. Brar, Richdeep S. Gill, Sumeet S. Gill, Haili Wang

**Affiliations:** a Department of Family Medicine, University of Calgary, Calgary, Alberta, Canada; b Department of Surgery, University of Alberta, Edmonton, Alberta, Canada; c Department of Kinesiology, University of Calgary, Calgary, Alberta, Canada

## Abstract

Meckel’s diverticulum is the most frequent congenital malformation of the gastrointestinal tract. The most common ectopic tissue within a Meckel’s is heterotopic gastric mucosa, with bleeding being the most common presentation. However, perforation of a Meckel’s diverticulum is a rare presentation. NSAID-associated Meckel’s perforation has been suggested, however has not been documented in the literature. We present a case of a 17-year-old female with acute abdominal pain and tenderness following a 24-hour history of excessive ingestion of NSAIDS for pain related to tooth extraction. Chest radiograph demonstrated free intra-abdominal air and she was subsequently taken for exploratory laparotomy. A perforated Meckel’s diverticulum was isolated and resected. Though NSAID-associated bleeding of heterotopic gastric mucosa has been described, and perforation of the Meckel’s diverticulum has been suggested, no clear association between heterotopic gastric mucosa and perforation exists. On the other hand, the relationship between NSAIDS and gastric ulcer perforation is well documented. A similar mechanism may also play a role in NSAID-associated Meckel’s perforation. With how common NSAID use is, we believe it is important to document NSAID-associated perforation of a Meckel’s diverticulum.

**Keywords:**

Meckel’s diverticulum; NSAIDS; Perforation; Heterotopic gastric mucosa

## Introduction

Meckel’s diverticulum is the most frequent congenital malformation of the gastrointestinal tract. It is present in 1% - 2% of the population and is due to the failure of the vitello-intestinal duct to obliterate [1, 2]. The most common presentations of symptomatic Meckel’s diverticulum are obstruction, bleeding and diverticulitis [3]. According to Park et al, 43% of the 1476 patients with Meckel’s diverticulum contained ectopic tissue [3]. The most common ectopic tissue was heterotopic gastric mucosa [3]. Heterotopic gastric mucosa was contained in 78% of bleeding diverticula [3], however causal relation to non-steroidal anti-inflammatory drug (NSAID) use remains unproven [4, 5]. Perforation is a very rare presentation of Meckel’s diverticulum, usually secondary to foreign objects or diverticulitis [6]. NSAID-associated Meckel’s perforation has been suggested, however has not been documented in the literature. We present a case of Meckel’s perforation associated with excessive use of NSAIDS.

## Case Report

**Figure 1.**
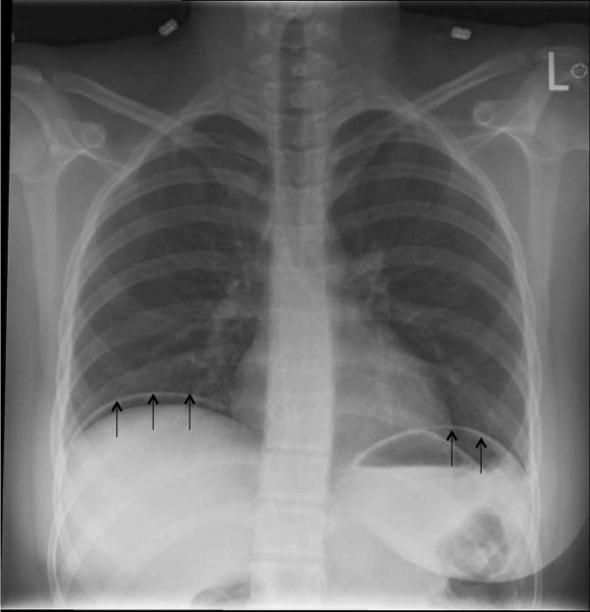
Chest radiograph demonstrating free intra-abdominal air below the diaphragm. (Black arrows demonstrate the air below the diaphragm).

A 17-year-old previously healthy female presented to her local health center with severe diffuse abdominal pain and bilateral shoulder tip pain. She was transferred to a tertiary level hospital for surgical consultation. The patient described progressively worsening abdominal pain over four days, and black vomiting starting earlier in the day. She stated the pain started 24-hours after having a tooth extraction, for which she was taking large quantities of NSAIDS. On examination, the patient was tachycardic with a low-grade fever. Abdominal examination revealed diffuse peritonitis. Her laboratory test revealed a mild leukocytosis, with no other abnormalities. A subsequent chest radiograph revealed free air under the diaphragm bilaterally (Fig. 1). Arrangements were made for emergent laparotomy for suspected gastric ulceration secondary to NSAID use. Intra-operatively, no gastric perforation was found. Further intra-abdominal examination revealed a 0.5 cm perforation in a Meckel’s diverticulum. A small bowel resection with primary anastomosis was completed. Patient recovered without complication. Final pathology revealed heterotrophic gastric mucosa.

## Discussion

Meckel’s diverticulum is the most common true diverticulum (contains all layers of bowel) of the gastrointestinal (GI) tract. It occurs in approximately 1% - 2% of the population [1, 2], however only 4% - 16% of those that have it will have complications. About half (45% - 60%) of patients who become symptomatic will present by the age of 2, with the majority of cases of Meckel’s diverticulum being found within 60 cm of the ileocecal valve [5].

Meckel’s diverticulum occurs because of incomplete obliteration of the viteline duct during the 5th week of gestation [7]. The viteline duct connects the fetal midgut to the yolk sac and progressively narrows with complete obliteration by the 7th gestational week. If it fails to obliterate there is potential for a patent duct, umbilical polyp, Meckel’s diverticulum, duct cyst or fibrous cord with Meckel’s diverticulum being the most common abnormality (97%).

In adults, the most common presentation of Meckel’s diverticulum is obstruction. Diverticulitis can occur but it is less common than appendicitis as the neck of the diverticulum is usually quite wide and there is little lymphoid tissue surrounding it. The most common presentation of Meckel’s diverticulum in children is painless lower GI bleeding [5]. About 50% of Meckel’s diverticula contain ectopic mucosa, with gastric mucosa being most common [3]. GI bleeds occur because of heterotopic gastric mucosa causing peptic ulceration of the diverticulum. Maieron et al found heterotopic gastric mucosa in 4 of 10 patients with lower GI bleeding and Meckel’s diverticulum. Of these 4 cases, NSAID use had occurred in 3 patients [7]. Mathur et al described the first case of NSAID-associated GI bleeding from Meckel’s diverticulum in an adult [4]. Khosa et al described a similar case of NSAID-associated bleeding in a 6-year-old boy [8].

Perforation is a relatively rare presentation of Meckel’s diverticulum. The literature reports cases of perforation secondary to diverticulitis of the Meckel’s diverticulum. Less commonly, perforation may be due to foreign body, such as fish bones [9], chicken bones [10], or a button battery [11]. The most common cause of gastric perforation, Helicobacter pylori (H. Pylori) have been shown to be absent in most Meckel’s diverticulum [4, 12]. Furthermore, Chan et al report that H. Pylori were absent in Meckel’s diverticulum even when the stomach was colonized by H. Pylori [13]. Though peptic ulceration leading to perforation of the Meckel’s diverticulum has been suggested [14], no clear association between heterotopic gastric mucosa and perforation exists. On the other hand, the relationship between NSAIDS and gastric ulcer perforation is well documented. NSAIDS inhibit endogenous prostaglandin synthesis leading to gastric mucosal injury [15]. This mechanism may also play a role in NSAID-associated Meckel’s perforation, as in our patient. We present here the first documented case of NSAID-associated perforation of a Meckel’s diverticulum.
